# Hypertensive response to exercise and exercise training in hypertension: odd couple no more

**DOI:** 10.1186/s40885-017-0067-z

**Published:** 2017-06-02

**Authors:** Elisa Caldarone, Paolo Severi, Mario Lombardi, Stefania D’Emidio, Andrea Mazza, Maria Grazia Bendini, Massimo Leggio

**Affiliations:** 1 Physical Medicine and Neurorehabilitation Operative Unit, Salus Infirmorum Clinic, Rome, Italy; 2 Department of Medicine and Rehabilitation, Cardiac Rehabilitation Operative Unit, San Filippo Neri Hospital – Salus Infirmorum Clinic, Via della Lucchina 41, 00135 Rome, Italy; 3 Cardiology Division, Santa Maria della Stella Hospital, Orvieto, Italy

**Keywords:** Hypertension, Exercise, Prevention and management of cardiovascular disease

## Abstract

The diagnostic and prognostic implication of exaggerated blood pressure response to exercise has been controversial, with opinions ranging from a benign process to a harbinger of potential cardiovascular morbidity. Nonetheless, lowering of blood pressure and prevention of hypertension is in first instance preferable by lifestyle changes, and many studies have shown the inverse association between physical activity level and the incidence of cardiovascular diseases suggesting low aerobic fitness as a strong predictor for future cardiovascular disease and all-cause mortality in both healthy and cardiovascular disease patients, including those with hypertension.

Endothelial function, large artery stiffness and neurohormonal response are surely implicated both in the development of exaggerated blood pressure response to exercise and in the positive effect of physical exercise in the prevention and management of hypertension and cardiovascular disease in general.

In their interesting and well documented review published in this issue Kim and Ha broadly described the possible pathophysiologic mechanisms of exaggerated blood pressure response to exercise and its clinical implications: in this regard, a very interesting issue could be represented by the role of exercise training. In fact, there is an the ample evidence in the literature that physical activity could positively affect endothelial function, arterial stiffness, neurohormonal response and finally blood pressure levels both in healthy men and in hypertensive patients and so should be considered a very important element in the prevention and management of cardiovascular disease.

The diagnostic and prognostic implication of exaggerated blood pressure response to exercise has been controversial, with opinions ranging from a benign process to a harbinger of potential cardiovascular morbidity [1]. Nonetheless, lowering of blood pressure and prevention of hypertension is in first instance preferable by lifestyle changes, and many studies have shown the inverse association between physical activity level and the incidence of cardiovascular diseases suggesting low aerobic fitness as a strong predictor for future cardiovascular disease and all-cause mortality in both healthy and cardiovascular disease patients, including those with hypertension [2]. Although an important challenge is to increase the attention on strategies to maintain or improve fitness and to intensify and support the efforts to encourage physical activity, a large body of previous evidences demonstrated that an exaggerated blood pressure response to exercise is not an innocuous finding but associated with increased risk for cardiovascular events, and has been shown to be a predictor of future hypertension and risk of cardiovascular mortality [3]. Endothelial function, large artery stiffness and neurohormonal response are surely implicated both in the development of exaggerated blood pressure response to exercise and in the positive effect of physical exercise in the prevention and management of hypertension and cardiovascular disease in general; consequently, it seems mandatory to mutually promote early diagnosis/treatment of exaggerated blood pressure response to exercise in healthy subjects without hypertension and encourage physical activity in prevention, treatment, and control of all stages of hypertension [2, 4].

In their interesting and well documented review published in this issue Kim and Ha [5] broadly described the possible pathophysiologic mechanisms of exaggerated blood pressure response to exercise and its clinical implications, emphasizing that this phenomenon is not a benign one but a quite dangerous alarm, and also suggesting that even if it is still controversial whether to treat exaggerated blood pressure response to exercise and treatment strategy still remains uncertain its pathologic nature and its association with functional and structural impairment of left ventricle, future development of hypertension and increased cardiovascular events require further investigation. In this regard, a very interesting issue could be represented by the role of exercise training. In fact, there is an the ample evidence in the literature that physical activity could positively affect endothelial function, arterial stiffness, neurohormonal response and finally blood pressure levels both in healthy men and in hypertensive patients and so should be considered a very important element in the prevention and management of cardiovascular disease. Hence, further consensus on definition and method to measure exaggerated blood pressure response to exercise is required, and prospective studies with aggressive lifestyle modification in individuals with exaggerated blood pressure response to exercise are needed to evaluate the potential benefits.
